# Supplementary material for: Helminth-induced Th2 cell dysfunction is distinct from exhaustion and is maintained in the absence of antigen
Source: PLoS Negl Trop Dis. 2019 Dec 9;13(12):e0007908. doi: 10.1371/journal.pntd.0007908 (PMC6922449; doi:10.1371/journal.pntd.0007908)
Supplement: S1 Fig — (PDF) [file pntd.0007908.s001.pdf]

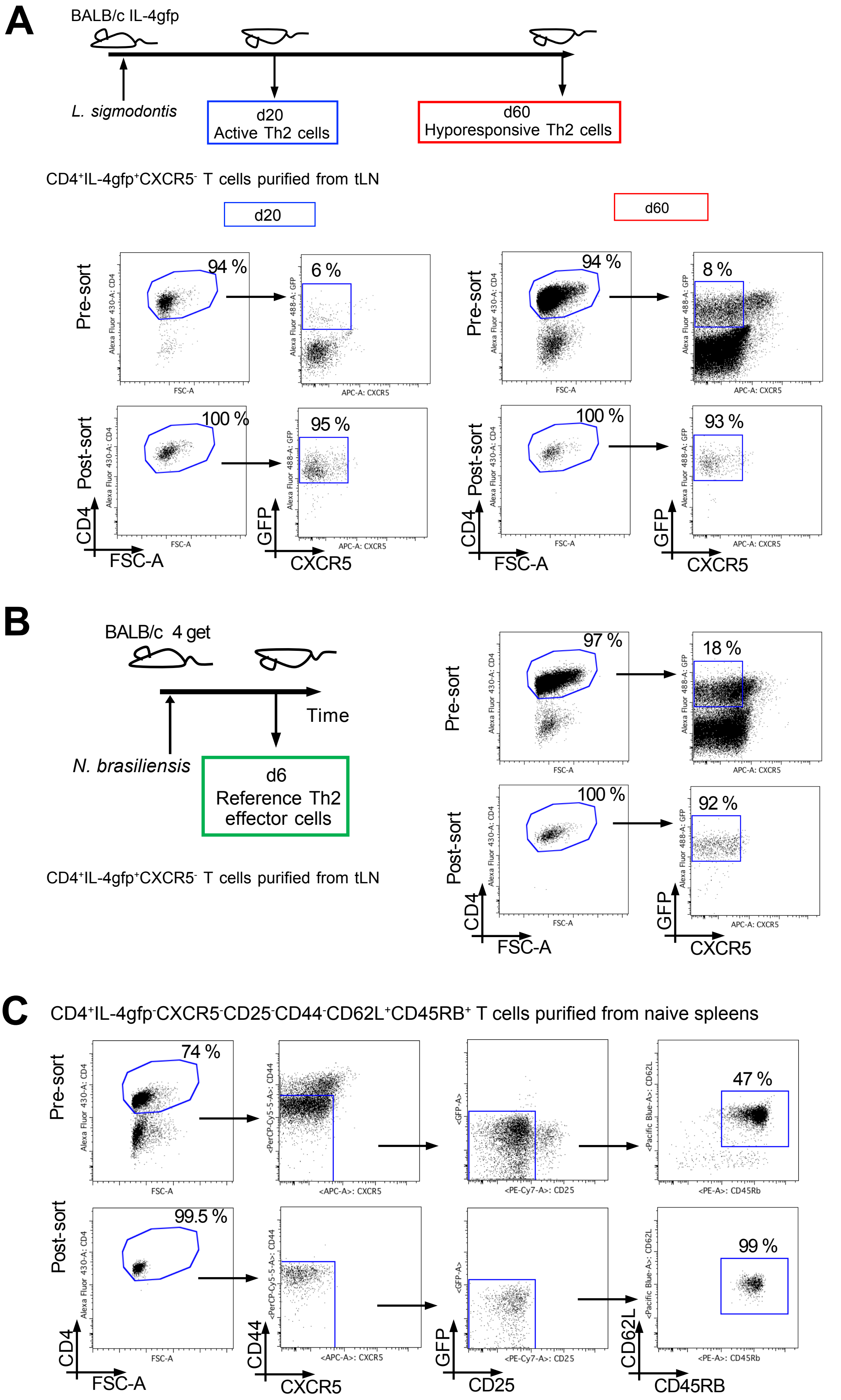

S1 Figure. Representative flow plots showing CD4$^+$IL-4gfp$^+$CXCR5$^-$ Th2 cell purifications from the tLN of *L. sigmodontis* (A) and *N. brasiliensis* (B) infected mice, and (C) CD4$^+$IL-4gfp$^-$CXCR5$^-$CD25$^-$CD44$^-$CD62L$^+$CD45RB$^+$ naive T cells from the spleens of uninfected mice. Percentages are calculated based on proportion of total viable cells.
