# Supplementary material for: Helminth-induced Th2 cell dysfunction is distinct from exhaustion and is maintained in the absence of antigen
Source: PLoS Negl Trop Dis. 2019 Dec 9;13(12):e0007908. doi: 10.1371/journal.pntd.0007908 (PMC6922449; doi:10.1371/journal.pntd.0007908)
Supplement: S2 Fig — (PDF) [file pntd.0007908.s002.pdf]

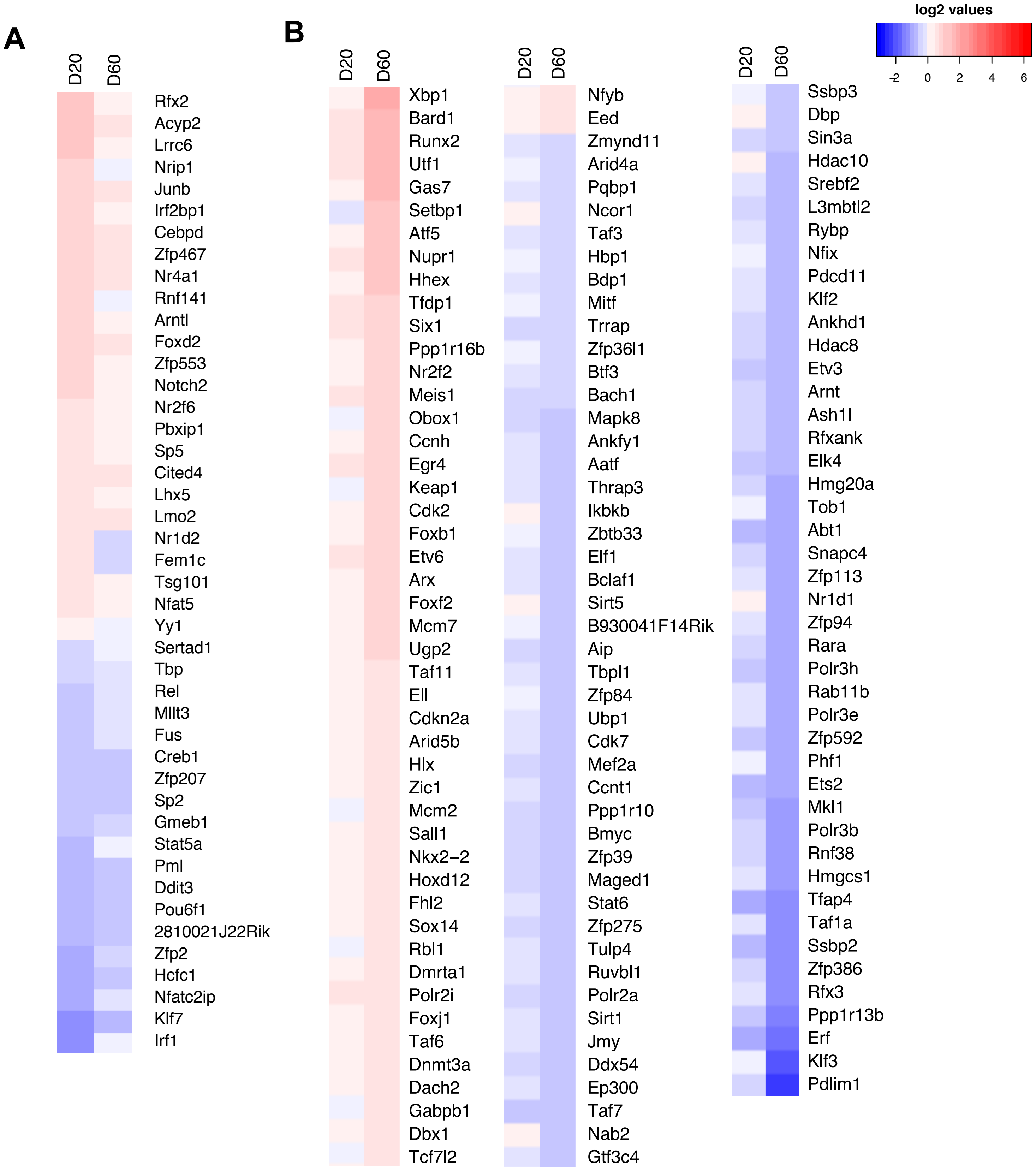


**S2 Figure. Transcription factors that are differentially expressed between d 20 and d 60 PleC IL-4gfp⁺ Th2 cells during *L. sigmodontis* infection.** (A) Heat map showing transcription factors that are significantly differentially expressed in d20 PleC IL-4gfp⁺ Th2 cells versus naïve T cells (adj. $p<0.05$), but not in PleC d60 IL-4gfp⁺ Th2 cells versus naïve T cells. (B) Heat map showing transcription factors that are significantly differentially expressed in PleC d60 IL-4gfp⁺ Th2 cells versus naïve T cells (adj. $p<0.05$), but not in PleC d20 IL-4gfp⁺ Th2 cells versus naïve T cells. Colours show fold increase (red) and fold decrease (blue) compared to naïve.
