# Supplementary material for: Helminth-induced Th2 cell dysfunction is distinct from exhaustion and is maintained in the absence of antigen
Source: PLoS Negl Trop Dis. 2019 Dec 9;13(12):e0007908. doi: 10.1371/journal.pntd.0007908 (PMC6922449; doi:10.1371/journal.pntd.0007908)
Supplement: S3 Fig — (PDF) [file pntd.0007908.s003.pdf]

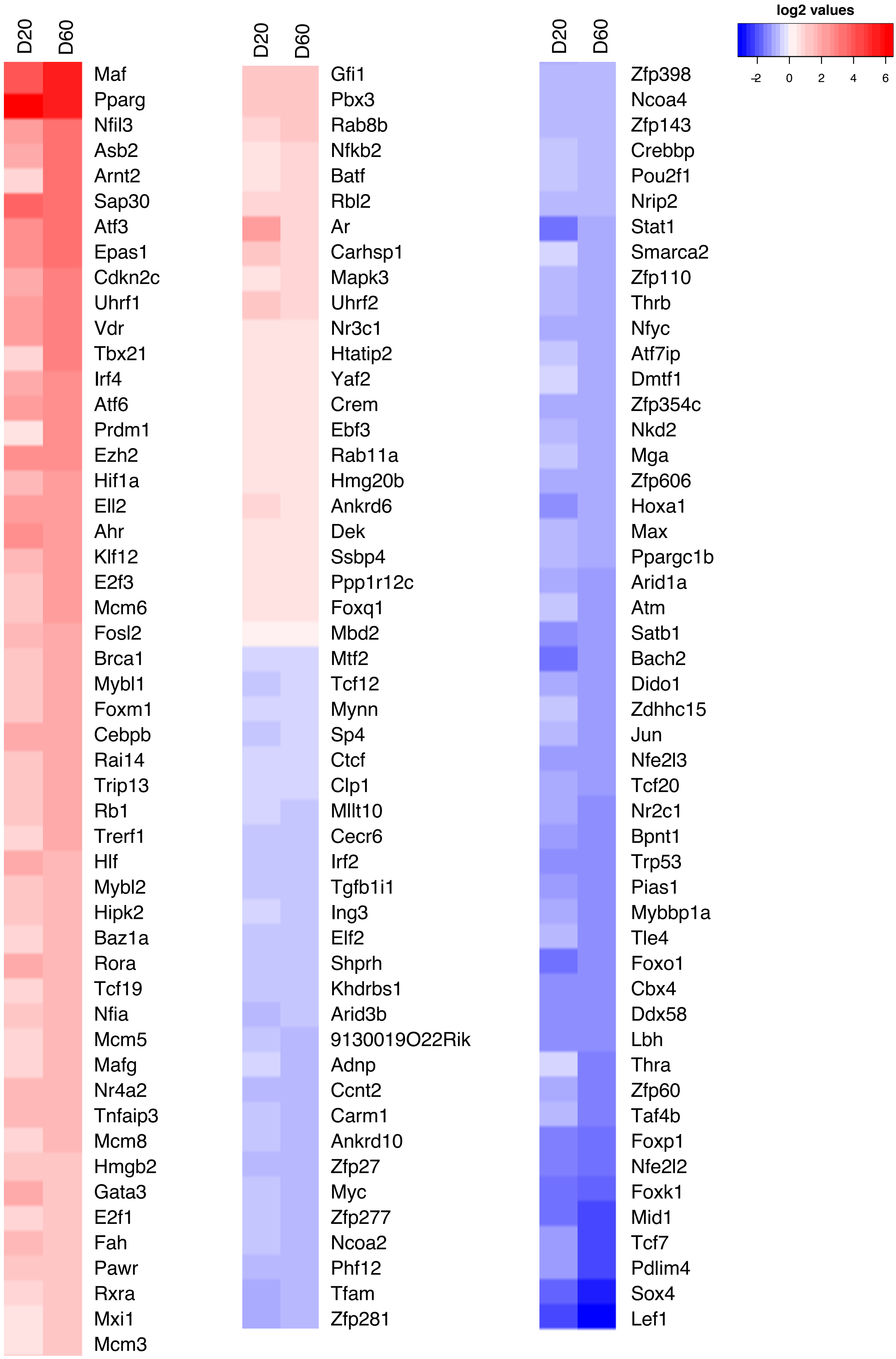

S3 Figure. Transcription factors that are expressed in common by d 20 and d 60 PleC IL-4gfp⁺ Th2 cells during *L. sigmodontis* infection. Heat map showing transcription factors that are significantly differentially expressed in both d20 and d60 IL-4gfp⁺ Th2 cells compared with naïve T cells (adj. p<0.05).
