# Supplementary material for: Helminth-induced Th2 cell dysfunction is distinct from exhaustion and is maintained in the absence of antigen
Source: PLoS Negl Trop Dis. 2019 Dec 9;13(12):e0007908. doi: 10.1371/journal.pntd.0007908 (PMC6922449; doi:10.1371/journal.pntd.0007908)
Supplement: S4 Fig — (PDF) [file pntd.0007908.s004.pdf]

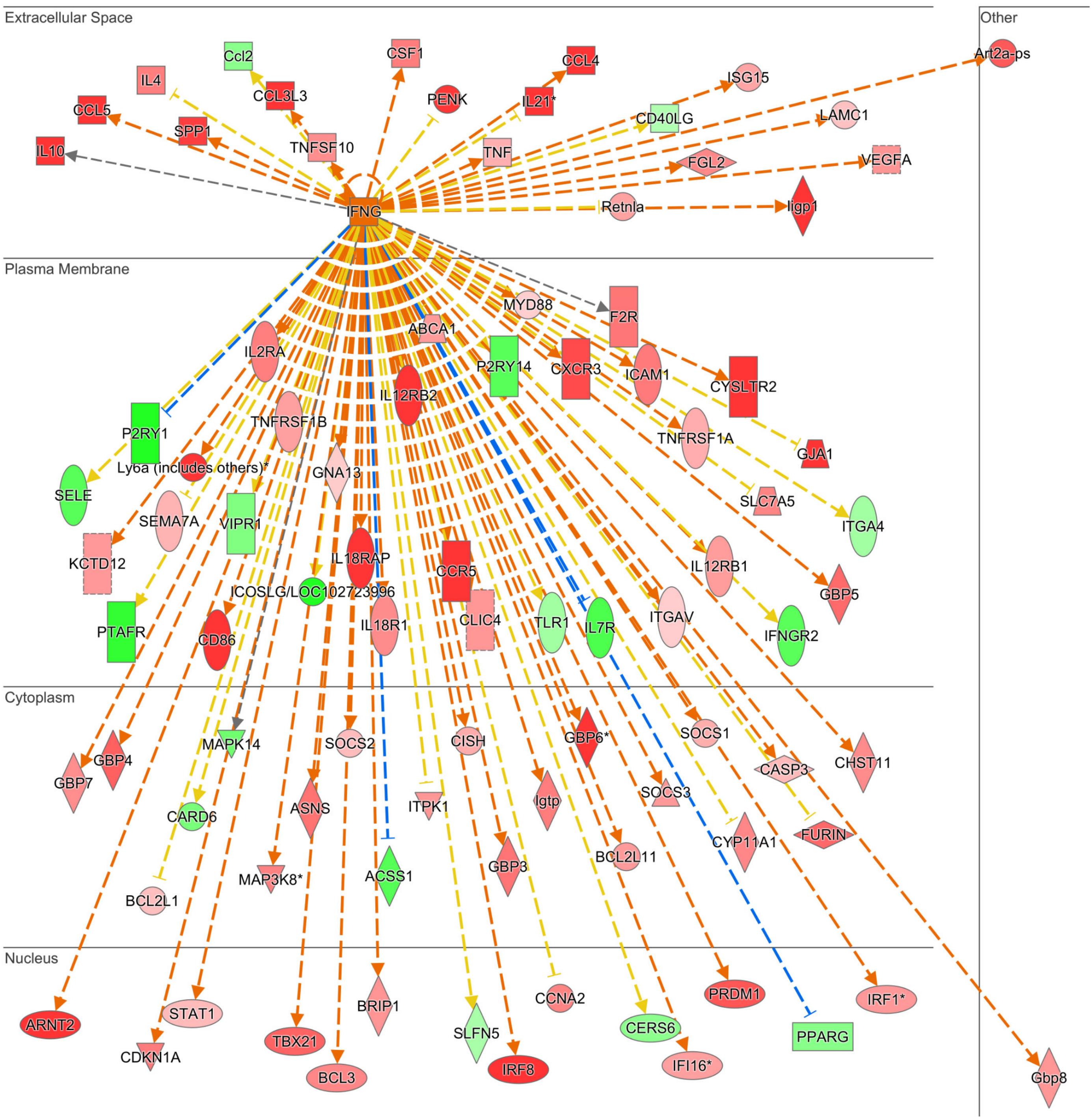

S4 Figure. IFN-γ is an upstream regulator of PleC d 60 IL-4gfp⁺ Th2 cells. IPA upstream regulator analysis based on genes that significantly (adjusted p<0.05) change in PleC IL-4gfp⁺ Th2 cells between d 20 and d 60 of *L. sigmodontis* infection. Red and green denote genes that are up- and down- regulated between d 20 and d 60 respectively, with colour intensity indicating magnitude of change. Lines show genes predicted by IPA to be increased (arrowheads) and decreased (closed end) in response to IFN-γ. Orange and blue lines indicate gene expression matches IPA prediction, whilst yellow lines indicate gene expression is inconsistent with IPA prediction. Shapes represent gene functions including cytokine (square, solid line), enzyme (vertical diamond), G-protein coupled receptor (vertical rectangle), transmembrane receptor (vertical oval), kinase (down triangle), peptidase (horizontal diamond), transporter (trapezoid), ion channel (vertical rectangle, dashed line), growth factor (square, dashed line), ligand-dependent nuclear receptor (horizontal rectangle), phosphatase (up triangle), transcription regulator (horizontal oval), and other (circle).
