# Supplementary material for: Helminth-induced Th2 cell dysfunction is distinct from exhaustion and is maintained in the absence of antigen
Source: PLoS Negl Trop Dis. 2019 Dec 9;13(12):e0007908. doi: 10.1371/journal.pntd.0007908 (PMC6922449; doi:10.1371/journal.pntd.0007908)
Supplement: S5 Fig — (PDF) [file pntd.0007908.s005.pdf]

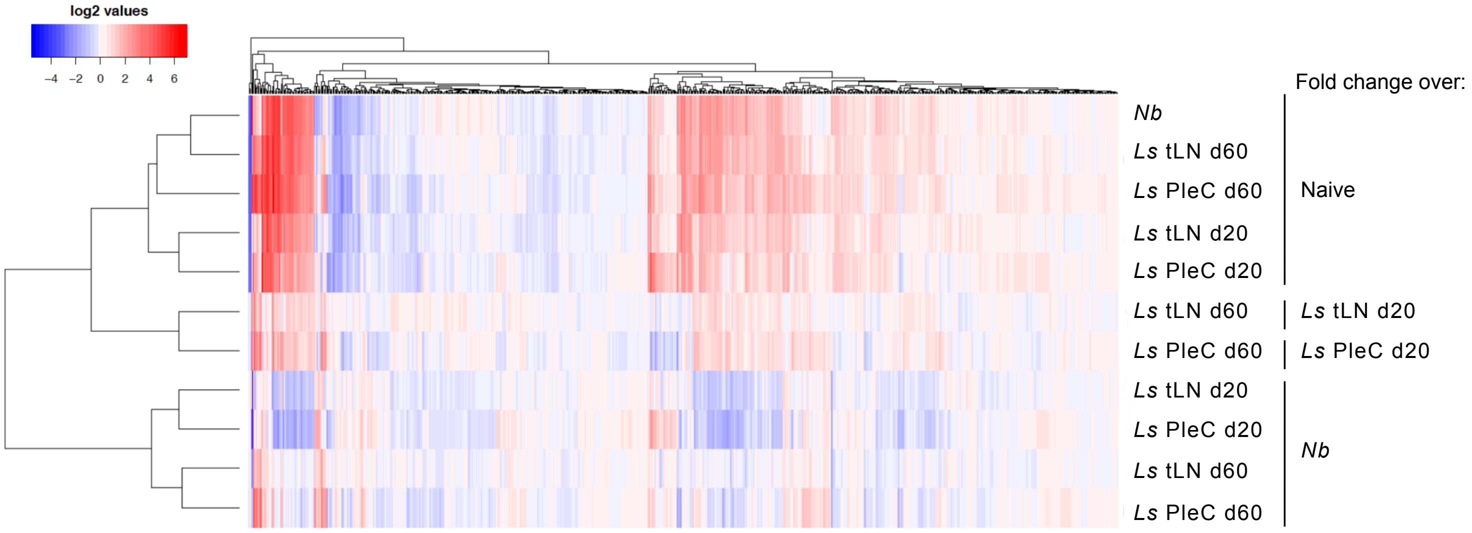

S5 Figure. Comparison of gene expression in peptide-induced tolerance and Th2 cell-intrinsic hypo-responsiveness. Unbiased hierarchical clustering of IL-4gfp^{+} Th2 cells purified from the PleC and tLN of mice infected with *L. sigmondontis* (*Ls*) and from the tLN of *N. brasiliensis* (*Nb*) infected animals. Clustering was performed based on Euclidean distance using the fold change of genes that are increased in CD4^{+} T cells following six rounds of peptide-induced tolerance that results in a stable unresponsive phenotype (Burton *et al.*, 2014, Nat. Comm. 5:4741).
