# Supplementary material for: Helminth-induced Th2 cell dysfunction is distinct from exhaustion and is maintained in the absence of antigen
Source: PLoS Negl Trop Dis. 2019 Dec 9;13(12):e0007908. doi: 10.1371/journal.pntd.0007908 (PMC6922449; doi:10.1371/journal.pntd.0007908)
Supplement: S1 Table — (DOCX) [file pntd.0007908.s007.docx]

| **Pathway ID** | **Pathway Name** | **PleC D60vN**  (adj. p value) | **tLN D60vN**  (adj. p value) |
| --- | --- | --- | --- |
|  | **Replication and Repair** |  |  |
| 3440 | Homologous recombination | 1.2e^-5^ | 2.9e^-5^ |
| 3430 | Mismatch repair | 2.0e^-3^ | 5.0e^-5^ |
| 3420 | Nucleotide excision repair | 6.9e^-3^ | 2.3e^-2^ |
|  | **Folding, sorting and degradation** |  |  |
| 4120 | Ubiquitin mediated proteolysis | 1.4e^-3^ | 1.9e^-3^ |
| 3018 | RNA degradation | 9.2e^-3^ | NS |
|  | **Metabolism** |  |  |
| 240 | Pyrimidine metabolism | 1.0e^-4^ | NS |
| 640 | Propanoate metabolism | 6.0e^-4^ | NS |
| 280 | Valine, leucine, and Isoleucine degradation | 8.7e^-4^ | NS |
| 450 | Selenocompound metabolism | 6.1e^-3^ | NS |
| 230 | Purine metabolism | 7.2e^-3^ | NS |
| 10 | Glycolysis/ gluconeogenesis | 8.1e^-3^ | NS |
| 562 | Inositol phosphate metabolism | 8.1e^-3^ | NS |
| 290 | Valine, leucine and isoleucine biosynthesis | 2.1e^-2^ | NS |
| 410 | Beta-alanine metabolism | 4.1e^-2^ | NS |
| 601 | Glycosphingolipid biosynthesis | 4.1e^-2^ | NS |
| 900 | Terpenoid backbone biosynthesis | 5.3e^-3^ | NS |
| 270 | Cysteine and methionine metabolism | NS | 1.0e^-2^ |
|  | **Signal Transduction** |  |  |
| 4070 | Phosphatidylinositol signaling system | 4.5e^-3^ | NS |
| 4150 | mTOR signalling pathway | 1.3e^-5^ | NS |
| 4010 | MAPK signalling pathway | NS | 1.0e^-4^ |
| 4620 | Toll-like receptor signalling pathway | NS | 8.9e^-3^ |
| 4664 | Fc epsilon R1 signalling pathway | NS | 2.1e^-2^ |
| 4012 | ErbB signalling pathway | NS | 3.8e^-2^ |
| 4370 | VEGF signalling pathway | NS | 4.4e^-2^ |
| 4621 | NOD-like receptor signalling pathway | NS | 4.8e^-2^ |

**S1 Table.** Table showing KEGG pathways enriched in PleC and/or tLN IL-4gfp^+^ Th2 cells at d 60 pi, but not at d 20 pi. NS = non-significant.
